# RING-Type E3 Ubiqitin Ligase Barley Genes (*HvYrg1–2*) Control Characteristics of Both Vegetative Organs and Seeds as Yield Components

**DOI:** 10.3390/plants9121693

**Published:** 2020-12-02

**Authors:** Zoltán Zombori, Bettina Nagy, Róbert Mihály, János Pauk, András Cseri, László Sass, Gábor Horváth V., Dénes Dudits

**Affiliations:** 1 Institute of Plant Biology, Biological Research Centre, Temesvári krt. 62., H-6726 Szeged, Hungary; zombori.zoltan@brc.hu (Z.Z.); nagy.bettina@brc.hu (B.N.); cseri.andras@brc.hu (A.C.); sass.laszlo@brc.hu (L.S.); 2 Doctoral School in Biology, Faculty of Science and Informatics, University of Szeged, H-6726 Szeged, Hungary; 3 Laboratory of Cellular Imaging, Biological Research Center, Temesvári krt. 62., H-6726 Szeged, Hungary; 4 Cereal Research Non-Profit Ltd., Alsó Kikötő Sor 9., H-6726 Szeged, Hungary; robert.mihaly@gabonakutato.hu (R.M.); janos.pauk@gabonakutato.hu (J.P.); 5 Institute of Genetics, Biological Research Centre, Temesvári krt. 62., H-6726 Szeged, Hungary; horvath.gabor@brc.hu

**Keywords:** *Hordeum vulgare* L., *HvYrg* gene variants, antisense, down regulation, shoot system, heading time, seed size, seed morphology, root development, digital imaging

## Abstract

Previously, studies on RING-type E3 ubiquitin ligases in cereals were preferentially focused on *GW2* genes primarily controlling seed parameters in rice and wheat. Here we report cloning two *HvYrg* genes from barley that share significant homology with rice *GW2* gene. In antisense genotypes efficiency of gene silencing varied between genes and transgenic lines: AS*HvYrg1*: 30–50% and AS*HvYrg2:* 20–27%. Reduced activity of both genes altered shoot system with increasing number of side shoots. Changes in leaf width, weight, or plant weight and height reached significant levels in some transgenic lines. Lowering expression of the two barley *HvYrg* genes caused opposite responses in spike development. Plants with AS*HvYrg1* gene construct showed earlier heading time and prolonged grain-filling period, while plants from AS*HvYrg2* genotype flowered in delay. Digital imaging of root development revealed that down-regulation of *HvYrg1* gene variant stimulated root growth, while AS*HvYrg2* plants developed reduced root system. Comparison of seed parameters indicated an increase in thousand grain weight accompanied with longer and wider seed morphology. In summary we conclude that in contrast to inhibition of *GW2* genes in rice and wheat plants, down-regulation of the barely *HvYrg* genes caused substantial changes in vegetative organs in addition to alteration of seed parameters.

## 1. Introduction

Insuring the yield potential and stability of small-grain cereals, such as wheat (*Triticum* species), rice (*Oryza sativa* L.), and barley (*Hordeum vulgare* L.) is a priority for global food security. Demand for cereals is expected to rise in coming decades because of increase in population and income, furthermore the continuous reduction of arable land worldwide. Currently, barley is the fourth most important cereal in the world, and barley grains are mainly used for animal feed and in the production of alcoholic beverages. Central objective of barley breeders is the creation of more adaptive varieties capable of achieving higher yields with less input requirements. Grain yield as the final end product of barley life cycle reflects metabolic functionality of plant organs in interaction with environmental factors. Overview of major yield determining genes in wheat and barley by [1] clearly shows complexity of developmental and physiological processes controlling plant and inflorescence architecture even seed characteristics. This publication identified different functional categories of barley genes encoding transcriptional factors influencing spikelet development, architecture and growth of inflorescence and grain number, and genes of cytokinin metabolism regulating plant architecture or grain number, furthermore, genes of gibberellin or brassinosteroid signaling and metabolism controlling plant architecture or culm (stem) length. The above mentioned list of yield-related barley genes can be extended with an additional one that was identified and characterized by Chinese research group [2]. This cloning work identified *HvYrg*1 gene from Tibetan barley that showed 86% identity with rice *GW*2 gene. Song et al. [3] cloned and characterized a new *GW2* QTL in rice that controls grain width and weight. The map-based cloning identified the *GW2* gene encoding a RING protein with E3 ubiquitin ligase activity. Sequencing the *GW2* allele from the *japonica* type rice variety, WY3, with a very large grain revealed a premature stop codon led to truncated protein. Using transgenic rice plants carrying antisense *GW2* cDNA the authors could demonstrate that the reduction or loss of function of *GW2* can result in increased grain width. These results focused the attention to ubiquitination as a key process in regulating seed size of crop plants.

Protein ubiquitination, including the sequential performances of ubiquitin-activating (E1), ubiquitin-conjugating (E2), and ubiquitin ligase (E3) enzymes targets specific substrates, and regulates localization, stability, and activity of modified proteins. Poly-ubiquitinated target proteins are degraded by the 26S proteasome complex. The RING-type E3 proteins are characterized by the presence of the RING domain, which is a cysteine-rich domain that co-ordinates two zinc atoms (see review [4]). The RING-finger domain acts as a protein–protein interaction domain and it is necessary to catalyze the ligase activity of RING-finger proteins being key regulators in phytohormone signaling or distinct plant developmental processes as well as abiotic stress responses [5,6]. Despite of the convincing results from studies on rice GW2 function a more complex picture can be outlined about the GW2 RING-type E3 proteins in other cereals. Li et al. reported two homologs of this gene, *ZmGW2-CHR4* and *ZmGW2-CHR5* in maize, and transcript abundance of maize *ZmGW2- CHR4* gene correlated negatively with kernel width in large number maize inbred lines [7]. Functional divergence of GW2 encoded pathways in cereals was highlighted by down-regulation of wheat *TaGW2* transcript-levels, which led to reduction in endosperm cell number associated with reduction in final grain weight and size [8]. These experimental results were not supported by other studies showing negative effects on expression of *TaGW2*-*A1*, a wheat homolog on thousand grain weight (TGW) [9,10,11]. In line with these studies, Zhai et al. have identified a new mutation as deletion in the promoter region of *TaGW2*-*A1* gene [12]. The reduced promoter activity and decreased expression caused increase in seed size. Constitutive expression of GW2 RING-type E3 protein genes was also recorded in vegetative tissues of cereal plants. In transgenic rice plants the *GW2* promoter strongly expressed the GUS reporter gene in leaves and roots [13]. In another set of transgenic rice plants GUS assay showed strong expression of *GW2* promoter in young panicle and anther [14]. Interestingly the loss of *GW2* function failed to alter plant height and flag leaf width in rice [4]. Based on these gene activity profiles, one can expect functional consequences of down regulation of *GW2* gene not only in growth and development of cereal seeds but also of vegetative organs.

In the present work, we include barley *(Hordeum vulgare* L.) as an additional cereal crop for extended functional analysis of RING-type E3 genes. First, we report identification of an additional variant (*HvYrg2)* of this yield-related gene family and present relation of the two barley proteins encoded by *HvYrg1* and *HvYrg2* genes to GW2 RING-type E3 proteins from other cereals. For functional characterization of these gene variants we generated antisense transgenic barley plants carrying amplified genomic DNA fragments including the RING-finger domain region. The T_2_ plants with reduced expression of *HvYrg1* and *HvYrg2* genes were used for quantification of phenotypic parameters of green organs, roots, and mature seeds. Despite a variable degree of gene silencing, the presented data support the conclusion that RING-type E3 ubiquitin ligases encoded by *HvYrg1* and *HvYrg2* are involved in regulation of both vegetative and generative organ size and developmental processes as timing heading or seed maturation in barley.

## 2. Results and Discussion

### 2.1. Two Homologs of HvYrg Genes Encoding RING-Type Protein as E3 Ubiquitin Ligases in Barley

**Figure 1:**
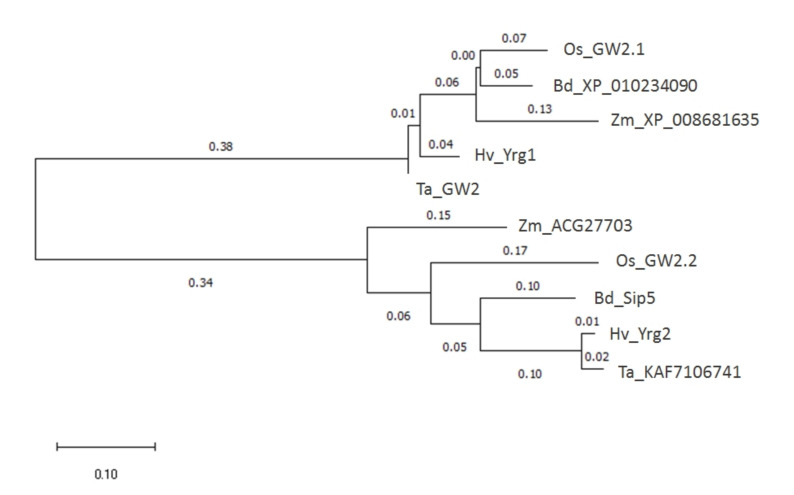
Phylogenetic tree of the *Yrg1 and Yrg2* genes in grass species.

Early discovery of the *GW2* gene encoding a RING-type protein as E3 ubiquitin ligase regulating seed size parameters in rice [3] has initiated wide research activities on different plant species including the major cereal crops [4,5]. The phylogenetic relations—based on their amino acid sequences—between the orthologs among the grass species are presented in Figure 1. The present work extends functional characterization of two orthologs of rice *GW2* gene from barley representing different genes with 62.1% identity at nucleotide level. They were annotated under gene name or accession numbers as *HvYrg1* (EU333863) and AK250398 as *HvYrg2* in the DDBJ/EMBL/GenBank Data Libraries. Amino acid sequence identity between the two RING protein encoded by the *HvYrg1* and *HvYrg2* genes was only 47.7% that can reflect functional divergence (see Supplementary Figure S1). Comparison of the two RING-finger domains revealed differences between these proteins at the conserved histidine (His) residue. The *HvYrg2* protein lacks this residue that is required for metal ligands (ML) to chelate two zinc atoms and define a cross-brace secondary structure for binding to the ubiquitin-conjugated E2 enzyme [4]. This structural characteristic was also recognized in two *Arabidopsis* RING-type E3 proteins (RING-C2 and Ring-v) as published by [15]. According to Song et al. because of the lack of a histidine residue these structures cannot be categorized as a RING domain [3].

### 2.2. Production of T_2_ Generation Transgenic Barley Plants with Silenced Expression of the HvYrg Gene Variants

Antisense transgene-mediated gene silencing systems are widely used in plant research as powerful reverse genetic methods for studying gene function [16]. In the present studies, we generated *HvYrg1* and *HvYrg2* transgenic barley lines constitutively expressing DNA fragments from *Yrg1* and *Yrg2* genes in antisense direction. The T_2_ progenies of the hygromycin resistant T_1_ plants were genotyped by PCR analyses. As shown by Table 1, transgenic barley plants from four AS*HvYrg1* and two AS*HvYrg2* lines were included into detailed characterization. Reduction in expression levels was higher (30–50%) in leaves of AS*HvYrg1* plants (except: AS*HvYrg1/1*: 20% reduction), than in leaves of AS*HvYrg2* plants (20–27%) as shown by Table 1. Similar range of silencing efficiencies was reported for the wheat *TaGW2* gene [8].

### 2.3. Altered Vegetative Organ Parameters in Transgenic Barley Plants with Silenced HvYrg Genes

Since antisense expression of the barley *HvYrg* gene fragments was based on the highly active constitutive ubiquitin promoter [17], we were able to analyze possible alterations in vegetative growth and organ sizes in addition to seed parameters. Here we report results of phenotyping from two different cultivation methodologies. In the first case the barley plants were grown in a traditional greenhouse, while the second experiment was carried out by using a semi-automated phenotyping platform for monitoring root traits [18,19]. As shown by Table 1, significant increase in flag leaf weight could be recorded in antisense transgenic plants from AS*HvYrg1/1*, AS*HvYrg1/2,* and AS*HvYrg2/1* lines. Silencing both HvYrg variants altered the shoot architecture. Golden Promise (GP) plants served as references produced preferentially primary shoots, while in the transgenic plants number of side shoots was higher than of main shoots (Table 1). These phenotypic responses may reflect changes in hormonal status as results of lowered activity of *HvYrg* genes. RING-type E3 ligases are important regulators of ABA and ethylene signaling [6]. In grasses, the axillary meristems are capable of giving rise to side tillers and the increased branching in transgenic barley plants may originate from a lowered dormancy effect of ABA. Several barley mutants (*granum-a (gra-a), grassy tillers (grassy), intermedium-c (int-c), many noded dwarf1 (mnd1),* and *many noded dwarf6* (mnd6) exhibit enhanced tillering [20]. In the present case, we can see an increased number of side shoots in transgenic barley plants with reduced expression of both *HvYrg* gene variants. In attempts to interpret global consequences of silencing the *HvYrg* RING-type E3 ligase genes, we have to consider relationships between seed and tillering parameters that is proposed by studies on several barley mutants [21]. At the end of growing period, silencing the variant *HvYrg1* gene caused 14–16% increase in biomass expressed as stem dry weight (Table 1). These alterations in plants from AS*HvYrg1/1*, and AS*HvYrg1/4* lines are in accordance with the higher tiller number and increased plant heights. In contrast to these observations, transgenic rice plants with antisense of *GW2* gene did not show alteration in plant height and flag leaf width [3]. Another type zinc finger gene (*NbZFP1*) encoding C3HC4-type RING finger proteins from *Nicotiana benthamiana* was silenced by VIGS technique without phenotypic changes [22].

### 2.4. Down-Regulation of Barley HvYrg Genes Modulates Growth and Developmental Characteristics of Transgenic Plants

**Figure 2:**
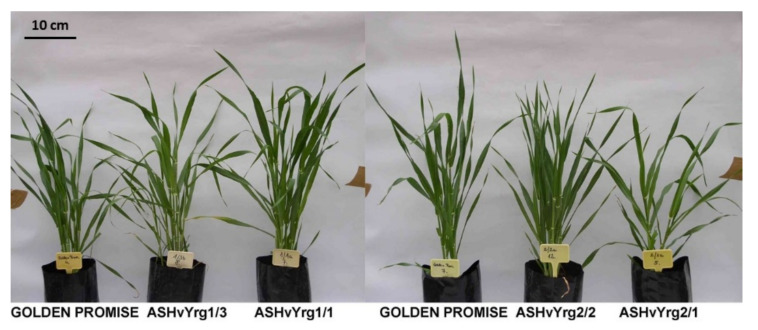
Characteristic differences between phenotypes of barley plants from the Golden Promise cultivar and transgenic lines with reduced of expression of *HvYrg* gene variants in vegetative growing phase.

Quantitative parameters presented in the Table 1, highlight essential changes in plant architecture as consequences of reduction in RING-type E3 ubiquitin ligase function. Figure 2 shows characteristic phenotypes of Golden Promise and antisense-transgenic plants at vegetative growth phase. Changes in shoot size and number can be recognized on the presented examples.

At vegetative growing phase, we could recognize increase in leaf width only in *ASHvYrg2* transgenic plants (Table 2). In contrast, *ASHvYrg1* plants developed leaves with similar wideness or narrower as the wild GP plants. These silencing effects can discriminate between the two *HvYrg* gene variants.

**Figure 3:**
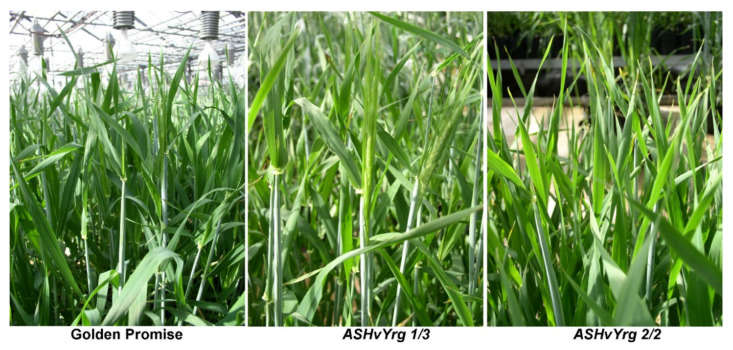
Altered timing in the spike development in transgenic AS*HvYrg 1/3* and AS*HvYrg 2/2* barley plants. Plants from the *ASHvYrg1/3* genotype show earlier heading time, while plants from the AS*HvYrg 2/2* genotype are delayed in flowering.

In later developmental phases, we monitored the induction of flowering and inflorescence development. Using reference Golden Promise plants, the down-regulation of the two barley *HvYrg* gene variants resulted in opposite responses. Spikes of plants from *ASHvYrg1* transformants emerged from the boot earlier within 5–7 days. In contrast, the *ASHvYrg2* plants showed delayed transition to flowering (Figure 3). Boden et al. [23] reported that the early flowering and vegetative growth phenotypes of the barley elf3 mutant can be related to gibberellin (GA) biosynthesis. The SCF type E3 ligases have been linked to GA pathway [6]. In rice, mutation in the Heading date Associated Factor 1 *(HAF1*) gene can cause a later flowering date. This factor was identified C3HC4 RING domain-containing E3 ubiquitin ligase [24].

**Figure 4:**
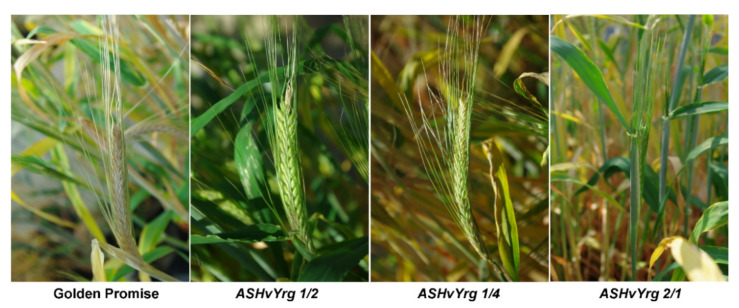
Alterations in spike development in barley plants with reduced activity of HvYrg gene variants. Down-regulation of expression of genes in plants from the AS*HvYrg2/1* and AS*HvYrg1/4* genotypes resulted in a prolonged grain-filling period, while the AS*HvYrg2/1* plants are late in spike development.

Alterations in activities of *HvYrg1* gene variant resulted not only in earlier heading dates, but also affected grain-filling duration. As shown by the Figure 4. despite of the earlier spike development, the grain-filling period was prolonged in AS*HvYrg 1/2* and AS*HvYrg 1/4* plants. In cereals, the duration of grain filling can influnce grain weight [25]. Since considerable reduction in *HvYrg1* gene expression was recorded in plants from these genotypes (Table 1), essential changes in the hormonal status of spike could be expected as consequence of the lowered RING-type E3 ubiquitin ligase function [6]. Spike development is an important factor in the yield production of barley that is under the control of hormonal crosstalk [26].

### 2.5. Down-Regulation of the HvYrg Gene Variants Can Differentially Modify Development of Root System Monitored in a Semi-Automated Phenotyping Platform

**Figure 5:**
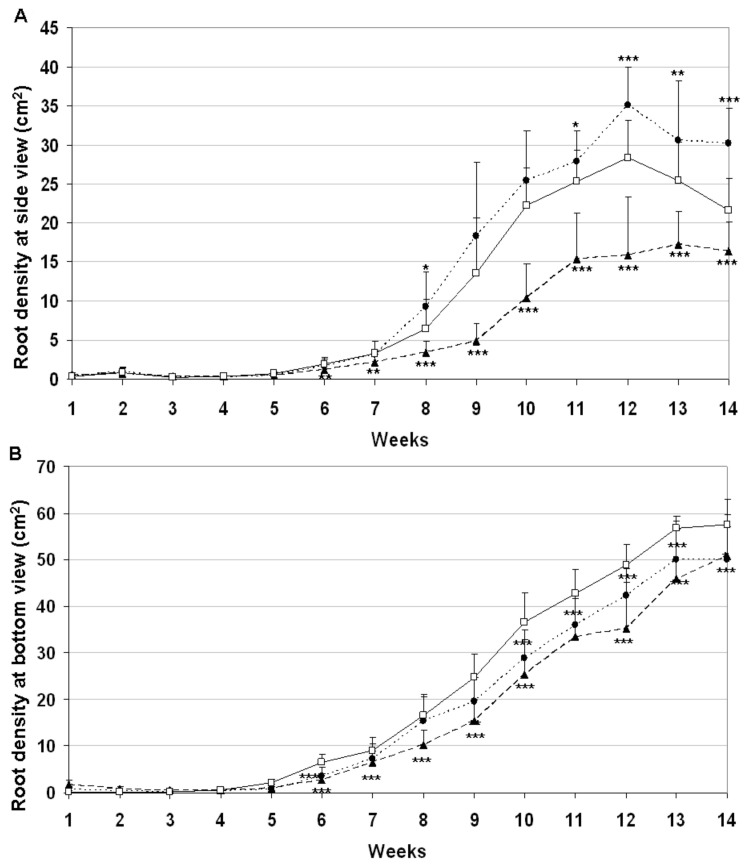
Silencing of barley *HvYrg* gene variants can differently alter development of root system monitored by digital imaging (AS*HvYrg 1/2* ▲, AS*HvYrg 2/1* ●, Golden Promise □). The mean ± SD were calculated from the data of 15 (PCR+) plants per genotype. Based on Student’s T-test, statistically significant events compared with Golden Promise mean value are indicated below with mean labels as *p* ≤ 0.001 ***, *p* ≤ 0.01 **, and *p* ≤ 0.05 *. (**A**): Average root density values calculated from white pixels generated by photography from four different side positions. (**B**): Antisense transformants of *HvYrg* gene variants show reduced root biomass accumulation at the bottom of plexiglass columns as recorded by digital imaging.

**Figure 6:**
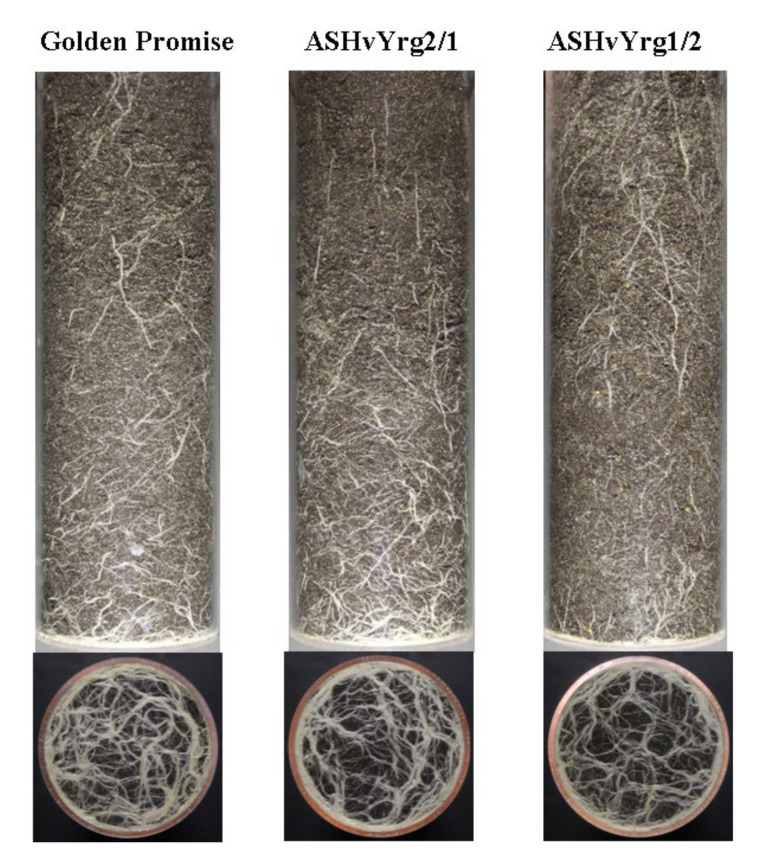
Characterization of root systems of barley plants with different activities of *HvYrg* gene variants by digital photography-based phenotyping. The diameter of the pots is 95 mm.

As an outcome of intensified plant phenotyping research, several alternative methods exist for the non-destructive imaging of root systems grown in either soil-free medium or rhizotrons filled with soil [27]. In the present study barley plants were grown in plexiglass columns that allowed to photograph the root system from four different side positions and from the bottom. The root-related white pixels were identified by subtracting the black soil background from the images. This methodology provided root density information about the development of roots during the growing period. As shown by Figure 5A, growth of root systems became intensified after eighth week of growing period with different rates between various genotypes according to side images. Down-regulation of AS*HvYrg2/1* gene variant stimulated root biomass in comparison to GP or AS*HvYrg1/2* plants. The later ones developed significantly reduced root system. Developmental differences can be seen in Figure 6 that also presents delay in growth of root system of AS*HvYrg1/2* plants. Root parameters were also quantified by images taken from the bottom side. Transgenic plants accumulated less root biomass at the bottom of plexiglass columns than the Golden Promise plants (Figure 5B).

As reviewed by [5] the RING-type E3 ligases play a key role in the control of different root systems. In rice plants overexpressing mutant EL5 proteins that are impaired in E3 activity exhibited rootless phenotype accompanied by cell death in root primordia [28]. The presented data provide additional insight into functional differentiation between the two barley *HvYrg* gene variants.

### 2.6. Silencing the HvYrg Barley Genes Can Alter Seed Parameters in Antisense Transgenic Plants

Transgenic barley plants carrying antisense constructs for *HvYrg1* gene variant were also characterized for seed production-related traits (Table 3). We selected this gene variant since in the T_2_ generation we could identify PCR+ and PCR− segregants. Data presented in the Table 3, shows comparison of these genotype categories. Considering the significant alterations in number of main and side shoots (Table 1) spike parameters were separately recorded in case of these different shoots. As number of kernels per spike is considered, silencing *HvYrg1* resulted in variable, transgenic line-dependent changes. The PCR+ plants from *HvYrg1/*1 line produced significantly more seeds per spike from the main branches than the PCR− variants. Similar trend could not be seen in the main branches of plants from *HvYrg1*/4 genotype. Reduction of kernel number/spike was characteristic for PCR+ plants from this genotype. Opposite, no significant changes could be seen in spikes from side shoots Comparison of this trait from PCR+ and PCR− plants of *HvYrg1/2* showed no difference between PCR+ and PCR− genotypes. Based on SD values, seed number/spike values exhibited considerable variation between plants from the same genotype. Reference data are available from studies on rice and wheat transformants with silenced *GW2* gene [3,8]. In wheat no significant differences in grain number per spike, while in rice *GW2* loss-of-function reduced the grain number per spike. Comparison of kernel weight/spike between PCR+ and PCR− plants presented increase in transgenic plants of the genotypes (AS*HvYrg1/2* and AS*HvYrg1/4*) with silenced *HvYrg1* gene. This alteration reached significant level only in the case of AS*HvYrg1/1* plants (Table 3). This trend cannot be seen on the spikes from the side tillers.

Of all the factors that influence yield, kernel weight measured as thousand kernel weight (TKW) is in the center of barley improvement. In the present study, we have recorded significant positive changes in this trait in main spikes of PCR+ plants from AS*HvYrg1/4* genotype compared to the PCR− segregants. Interestingly, side heads of AS*HvYrg1/2* and AS*HvYrg1/4* of barley produced larger seeds. The TKW values from side shoots were higher even in comparison to the Golden Promise seeds. Since in these transgenic plants the silencing effects were significant (see Table 1) we may postulate the negative regulatory role of *HvYrg1* gene in control of seed development. Increased TKW parameters were described for antisense *GW2* transformants of rice [3]. This modification was accompanied with reduction in grain number per main panicle. Similar trend can be seen in spikes of AS*HvYrg1/4* of barley plants.

**Figure 7:**
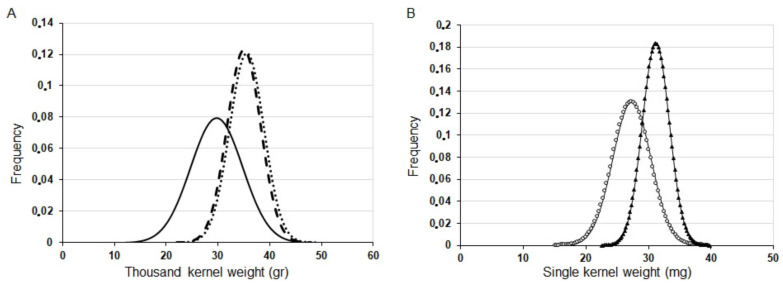
Representative kernel weight distribution for GP and AS transformants. (**A**): thousand kernel weight (g) in GP (____ $\bar{x}$ = 29.69 g); *ASHvYrg1/1* (……. $\bar{x}$ = 35.53 g); *ASHvYrg2/2* (- - -, $\bar{x}$ = 34.94 g). (**B**): differences in weight distribution of single kernels produced by the PCR+ plant (N° 13: $\bar{x}$ = 34.39 mg, 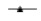) and PCR− plant (N° 10: $\bar{x}$ = 28.07 mg, 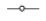) segregants of *ASHvYrg1/1* line.

As an additional grain parameter, the weights of single grains were also characterized. Because of the significant variation in this quantitative trait, we present distribution curves in addition to the average values. Figure 7A clearly shows larger kernels as shown by the TKW values for the transgenic plants carrying silenced variants of both *HvYrg* genes in T_2_ generation in comparison to GP kernels. The average TKW values from main and side spikes was 29.69 g for GP, 35.53 g for *ASHvYrg1/1* and 34.94 g for AS*HvYrg2/2* plants. Detection of PCR+ and PCR− segregants in the same plant population allowed direct comparison of phenotypic differences caused by the reduction of gene expression. Figure 7B shows a shift of the distribution curve towards larger kernels for N° 8 plants with silenced *HvYrg1* gene expression in comparison to the PCR− N° 3 plants.

Since increase in kernel weight of NIL(GW2) in rice plants was primarily due to increased grain width, followed by grain thickness and length [3], we also quantified the seed size parameters in barley AS genotypes. As described in the Material and Methods grain length and width parameters were quantified by the Seed Size Analysis Program v0.95. Pixels for the color of seed surface were used for prediction of seed size. As shown by Table 4 grains from AS*HvYrg1/3,* AS*HvYrg1/4,* and AS*HvYrg2/1* lines were significantly longer than the Golden Promise kernels. We also measured the length of individual grains (Supplementary Figure S2). Frequency of longer kernels was increased in the case of AS*HvYrg1/4* plants.

In agreement with ASGW2 transgenic rice seeds [3] the wideness of kernels from three barley transformed lines (AS*HvYrg1/3;* AS*HvYrg1/4,* and AS*HvYrg2/1*) was also increased according to pixel based calculation (Table 4). Plants from the AS*HvYrg1/2* genotypes showed significant reduction in this trait.

According to data of single kernels produced by plants from AS*HvYrg1/1* and AS*HvYrg2/2* lines developed wider kernels than of GP plants (Supplementary Figure S3). Comparison of kernels from single PCR+ and PCR− plants showed similar alteration in this trait (Figure 7B). In interpreting these alterations, we can rely on studies on rice ASGW2 transgenics [3]. The outer parenchyma cell layer contained substantially more cells, while endosperm cells showed increased size without changes in the cell number. The presented ASGW2 transgenic plants can serve as an experimental material for future studies on involvement of RING-type E3 ligases in cell division control in plants.

Despite of variable efficiency in silencing of *HvYrg* gene variants, the present study focuses the attention on a central role of RING-type E3 ligase pathway in controlling cereal plant architecture, growth and development. The presented alterations detected in phenotypic traits of antisense barley plants are similar to those described in studies on seed characteristics of rice and wheat plants with additional insight to response of vegetative organ including shoot and root system. Role of *HvYrg* gene variants in regulation of heading time and grain filling period is supported by this analysis. The present use of constitutive promoter allowed to gain basic functional information, but it can be limiting factor by generating multiple alterations in different traits in a breeding project. Therefore, based on the presented results, it is advisable to induce gene specific mutations by genome editing tools for improvement of agronomic traits.

## 3. Materials and Methods

### 3.1. Construction of Antisense HvYrg RING-Type E3 Expression Vectors and Barley Transformation

Genomic DNA samples were isolated with a CTAB-based extraction method according to [29] from “Golden Promise” (GP) barley variety. BLAST analysis was carried out to identify the barley orthologues of *GW2* RING-type E3 genes (*HvYrg1* and *HvYrg2*) in the barley genome sequence data base [30]. The presented primer sequences amplified 512 bps of *HvYrg1 (Yrg1_*Forward *5′-GGGAGCTTTATGCCTTTTGAGCAACC-3′*, Reverse*5′-GTGTGCGTTCTACCATGAGCTTCTGC-3′*) and 539 bps of *HvYrg2 (Yrg2_*Forward *5′-ATAGGTGCCGTGCCACCAACAC-3′*, Reverse *5′-TACCGCCAAGCTAACGCTGGAG-3′*) fragments. In an additional PCR cycle, these fragments were extended by *Spe*I and *BamH*I restriction sites. The fragments were digested with the appropriate enzymes (Thermo Scientific, Waltham, USA) and were cloned at *BamH*I and *Spe*I sites in the first cloning site of pUbi-AB intermediate vector [31]. This expression cassette was transferred into the p6d35s binary plant expression vector [32] with double enhanced *CaMV35S* promoter using *Sfi*I (New England Biolabs, Hitchin, Hertfordshire, UK) digestion and ligation [31]. Plasmid DNA was purified with GenElute™ HP Plasmid Miniprep Kit (Sigma, St. Louis, MO, USA) and the nucleotide sequences of the *GW2* RING-type E3 gene constructions were determined by the ABI 3100 Genetic Analyzer from Applied Biosystem (Foster City, CA, USA).

For the stable transformation of immature barley embryos (GP) we used the LBA4404 *Agrobacterium tumefaciens* strain by following protocol from [33]. Transgenic barley plants were tested for the integration of GW2 Ring-type E3 gene fragments and hygromycin resistance genes (Forward *5′- CCTGAACTCACCGCGAC -3′*, Reverse *5′- GCTCATCGAGAGCCTGC -3′*). We were able to establish four independent transgenic lines producing T2 generation plants with the AS*HvYrg1* construct and two lines with the AS*HvYrg2* constructs.

### 3.2. Growth Conditions for Barley Plants in Greenhouse or Semi-Automatic Phenotyping Platform

Regenerated hygromycin resistant plantlets were cultured in vitro and later transferred into soil in greenhouse. The T_1_ seeds were collected and sown into plastic pots (diameter 16 cm) containing a mixture of soil and sand (2:1, *v*/*v*) under a cycle of 12 h illumination (250 mmol m_2 s_1)/12 h dark. The T_2_ plants from the analyzed transgenic lines were tested for the presence of *HPT* selective marker gene and the *HvYrg Ring-type E3* gene fragments in antisense orientation.

At the end of vegetative growing period, we measured parameters as plant height, dry biomass of whole plants, and of three flag leaves. After harvesting, we determined seed number and weight per spike, furthermore thousand kernel weight (TKW). For comparative quantification of individual seeds (seed size, length, and width) we used pixel-based imaging. From each genotype, 80–80 seeds were measured individually with Ohaus Model EP114C analytical balance by 0.1 mg accuracy. Seed parameter analysis of the same samples was performed using the Seed Size Analysis Program (SSAP) v. 0.95 of the Plant Complex Stress Diagnostic System developed by László Sass at the Biological Research Centre, Szeged.

For characterization of root growth of barley plant from GP and *ASHvYrg1* and *ASHvYrg2* lines we used a semiautomatic phenotyping platform described previously [18,19,34]. The T_2_ seeds were sown into radio-tagged plexiglass columns with a mixture of 80% Florimo peat soil and 20% sandy soil. Five plexiglass columns surrounded with polyvinyl chloride tubing were placed on a metal rack. Three racks were used for each genotype with random arrangement. In the case of roots, the plexiglass columns were photographed from four different side positions and from the bottom. The root-related white pixels were identified by subtracting the black soil background from the images. Pixel numbers were converted to millimeters using 95-mm diameter pots captured in the images. To characterize the root area appearing at the surface of the chamber, the metric values of the area of the four side view projections (90° rotation) are summarized and the metric value of the area of the bottom view.

### 3.3. Quantitative Real-Time PCR (qRT-PCR) for Measurement of Expression of HvYrg1 and HvYrg2 Gene Variants in Transgenic Barley Plants

The total RNA samples were isolated from young leaves of two individuals of GP and T_2_ generation barley plants according to the AGPC (acid guanidinium thiocyanate-phenol chloroform) method [35]. For the cDNA synthesis 1 µg of total RNA was used, following the First Strand cDNA Synthesis Kit manual (Fermentas Life Sciences, Vilnius, Lithuania). For the quantitative RT-PCR reactions we used the following primers: ASHvYrg1_Forward *5′-TGCAGCACATCCTATTCAGC-3′,* Reverse *5′-GAATGGAAAGACCGCATGTT-3′*, ASHvYrg2_Forward *5′-GTTTCCTCTTGTGCGTGACA-3′*, Reverse *5′-TATCCCACCAGTCCCTATGC-3′.*

These were designed by the Primer Express Software from Applied Biosystems (Foster City, USA). The qRT-PCR reactions were carried out in the ABI PRISM 7000 Sequence Detection System using the SYBR Green PCR Master Mix and the reaction conditions were the same as described by [36]. The 2^−ΔΔC(T)^ method was used to analyze the real-time PCR data [37] and the expression of our examined genes was normalized to the reference gene (18S RNA, Forward *5′-GTGACGGGTGACGGAGAATT-3′,* Reverse *5′-GACACTAATGCGCCCGGTAT-3′).*

### 3.4. Data Management

The statistical significance of the results was determined using the Microsoft Excel 2003 software (Microsoft Inc., Redmond, WA, USA) Student’s *T*-test.

## Figures and Tables

**Table 1:** Reduced expression of *HvYrg* gene variants in leaves of antisense transgenic barley plants and parameters of vegetative traits. The mean ± SD were calculated from the data of 9–20 plants per genotype. Based on Student’s T-test, statistically significant events compared with Golden Promise (GP) mean value are indicated below with mean labels as *p* ≤ 0.001 ***, *p* ≤ 0.01 ** and *p* ≤ 0.05 *.

Genotype		Golden Promise	ASHvYrg 1/1	ASHvYrg 1/2	ASHvYrg 1/3	ASHvYrg 1/4	ASHvYrg 2/1	ASHvYrg 2/2
Transcription	Normalized	1	0.86	0.49	0.69	0.49	0.73	0.80
Weight of 3 Flag Leaves (g)	MEAN	0.15	0.19 ***	0.19 **	0.17	0.17	0.25	0.15
	SD	0.003	0.04	0.04	0.05	0.06	0.07	0.04
	*p*-value		<0.001	0.001	0.111	0.085	0.75	0.638
Shoot Number (main)	MEAN	4.33	3.64	2.5	2.44 ***	2.31	1.25 ***	2.68
	SD	0.98	1.45	0.798	1.54	1.5	0.46	1.15
	*p*-value		0.849	0.666	<0.001	0.057	<0.001	0.75
Shoot Number (side)	MEAN	1.88	2.50	3.58 **	5.39 ***	4.38 ***	3.62 **	3.33 **
	SD	0.27	1.30	1.16	2.93	2.06	1.06	1.37
	*p*-value		0.208	0.0037	<0.001	<0.001	0.004	0.005
Plant Weight (g)	MEAN	9.02	11.02 **	8.90	8.61	10.17 *	9.38	9.03
	SD	0.92	1.39	1.07	1.76	2.05	1.41	1.7
	*p*-value		0.005	0.32	0.369	0.034	0.456	0.907
Plant Height (cm)	MEAN	65.06	70.47 **	60.92	70.50	70.75 *	67.52	55.88
	SD	11.26	6.21	4.70	7.25	7.30	7.67	7.74
	*p*-value		0.013	0.155	0.087	0.033	0.86	0.758

**Table 2:** Down-regulation *of HvYrg2* barley gene variant can result in development of wider leaves. The mean ± SD were calculated from the data of 15 plants per genotype. Based on Student’s T-test, statistically significant events compared with Golden Promise mean value are indicated below with mean labels as *p* ≤ 0.001 ***.

	Leaf Width (mm)		
Genotypes	Mean	SD	*p*-Value
Golden Promise	9.99	0.73	-
ASHvYrg1/2	9.94	0.57	0.21
ASHvYrg1/4	8.94	0.57	0.16
ASHvYrg2/1	11.30 ***	0.77	<0.001
ASHvYrg2/2	11.69 ***	0.87	<0.001

**Table 3:** Comparison of grain yield-related traits from transgenic (PCR+) and non-transgenic (PCR−) segregants in T2 generation of antisense transformation of *HvYrg1* gene of barley. The mean ± SD were calculated from the data of 14 (PCR+) and 5 (PCR−) plants per genotype. Based on Student’s T-test, statistically significant events compared with Golden Promise mean value are indicated below with mean labels as *p* ≤ 0.001 ***, *p* ≤ 0.01 ** and *p* ≤ 0.05 *.

Genotypes	Origin of Spikes		Kernel No/Spike			Kernel Weight/Spike (g)			Thousand Kernel Weight (g)		
			Mean	SD	*p*-Value	Mean	SD	*p*-Value	Mean	SD	*p*-Value
Golden Promise	main		29.30	2.63		0.95	0.07		32.85	2.6	
	side		23.50	4.03		0.47	0.11		21.26	3.25	
ASHvYrg1/1	main	PCR+	28.47 *	2.27		0.94 *	0.07		33.01	2.76	
		PCR−	26.00	1.87	0.039	0.85	0.05	0.017	33.09	3.77	0.962
	side	PCR+	19.57	4.86		0.54	0.14		27.8 ***	3.80	<0.001
		PCR−	21.16	3.28	0.514	0.62	0.07	0.314	29.64	2.01	0.340
ASHvYrg1/2	main	PCR+	30.44	2.28		0.90	0.10		29.81	3.58	
		PCR−	30.50	0.94	0.958	0.84	0.02	0.170	27.85	0.47	0.248
	side	PCR+	25.73	2.05		0.57	0.11		26.39 ***	4.50	0.002
		PCR−	25.03	2.72	0.56	0.55	0.07	0.680	22.20	2.11	0.046
ASHvYrg1/4	main	PCR+	22.64 *	4.08		0.77	0.09		32.07 *	3.90	
		PCR−	28.13	0.01	0.012	0.78	0.04	0.803	28.09	0.87	0.04
	side	PCR+	18.16	4.91		0.48	0.14		28.87 ***	4.94	<0.001
		PCR−	21.16	3.38	0.24	0.54	0.06	0.395	24.10	5.57	0.094

**Table 4:** Comparisons of kernel width and length from barley genotypes based on the highest values (*n* = 20) after imagining of grains. Based on Student’s T-test, statistically significant events compared with GP mean value are indicated below with mean labels as *p* ≤ 0.001 *** and *p* ≤ 0.05 *.

	Kernel Length (mm)			Kernel Width (mm)		
Genotypes	Mean	SD	*p*-Value	Mean	SD	*p*-Value
Golden Promise	10.95	0.31		3.69	0.1	
ASHvYrg1/2	10.77	0.27	0.051	3.57 ***	0.08	<0.001
ASHvYrg1/3	11.18 *	0.37	0.041	3.85 ***	0.12	<0.001
ASHvYrg1/4	12.66 ***	0.57	<0.001	4.03 ***	0.15	<0.001
ASHvYrg2/1	11.62 ***	0.34	<0.001	3.93 ***	0.12	<0.001

## Supplementary Materials

The following are available online at https://www.mdpi.com/2223-7747/9/12/1693/s1.
