# Supplementary material for: RING-Type E3 Ubiqitin Ligase Barley Genes (HvYrg1–2) Control Characteristics of Both Vegetative Organs and Seeds as Yield Components
Source: Plants (Basel). 2020 Dec 2;9(12):1693. doi: 10.3390/plants9121693 (PMC7761584; doi:10.3390/plants9121693)
Supplement: Supplementary file 1 [file plants-09-01693-s001.zip › Zip_supplementary/Supplementary data.docx]

**Supplementary data**

HvYrg1 1 MGNRIGGRR-KAGVEERYTRPQGLYEHRDIDQKKLRKLILETKLAPCYPGADDAAGADLE
HvYrg2 1 MGNRVGGRRRRPPVDERYTQPQGLYPHPDIDLKKLRRLILEAKLAPCHPGADDP-RPDLD


HvYrg1 60 ECPICFLYYPSLNRSKCCSKGICTECFLQMKPTHTARPTQCPFCKTPNYAVEYRGVKTKE
HvYrg2 60 ECPICFLFYPSLNRSKCCAKGICTECFLQMKSPTSCRPTQCPYCKMLNYAVEYRGVKTKE


HvYrg1 120 ERSIEQFEEQKVIEAQMRMRQQALQDEEDKMRRKQSRCSSSRTIAPTTEVEYRDICSTSY
HvYrg2 120 EKGVEQIEEQRVIEAQIRMRHQELQDDAERLKNKQVAASTDEVTTA--RV---EPCDTGG


HvYrg1 180 SAPPYRCTEQETECCSSEPSCSAQANM----RSFHSRHTRDGNIDMNIEDMMVMEAIWRS
HvYrg2 175 TSTPAASGAQ----GNDAPSCQVQHSELLLKNAERLRQLRDNNFDMDLEEVMLMEAIWLS


HvYrg1 236 IQEQGSIGNPACGSFMPFEQPTRERQAFVAASPLEIPHPGGFSCAVAAMTEHQPSSMDFS
HvYrg2 231 VQDQEALGNPGSIGAVPPTLPLRCYDAS-GATSAEAAPPGGFACAVAALAEQQHMLGDPS


HvYrg1 296 YMTGS--SAFPV----FDMFRRPCNIAGGSLRAVESSLDSWSGIAPSGTRREMVREEGEC
HvYrg2 290 SAATCQTSRHDILSRSQRSFTEDLSIAGSSSSAIRVEE------PPSNGRT--PQARDYS


HvYrg1 350 SIDHWSEGAEAGTSYAGSDIMADAGTMPPLP-FADNYSMAASHFRPESIEEQMMYSMAVS
HvYrg2 342 NNDGWSDVAEASTSCAGSDVTVEAGAASLAAAAASDVSSIGSGNVPDSFEEQMMLAMALS


HvYrg1 409 LAEAHGRTH-TQGLTWL
HvYrg2 402 LVDARGVGGSPPALAWR


**Supplementary figure S1.** The alignment of the amino acid sequences of two barley YRG proteins. The black frame shows the differences between their RING-domains.


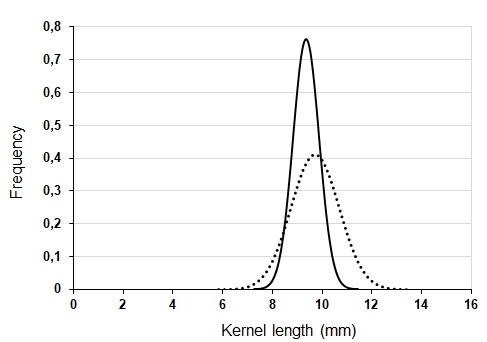


**Supplementary Figure S2.** Differences in distribution of length of grains produced by the PCR+ (*ASHvYrg1/4*  N^o^ 17; **…...,** **x̄ =** 9.70mm) and GP (**___**,**x̄ =** 9.35mm) plants.

**
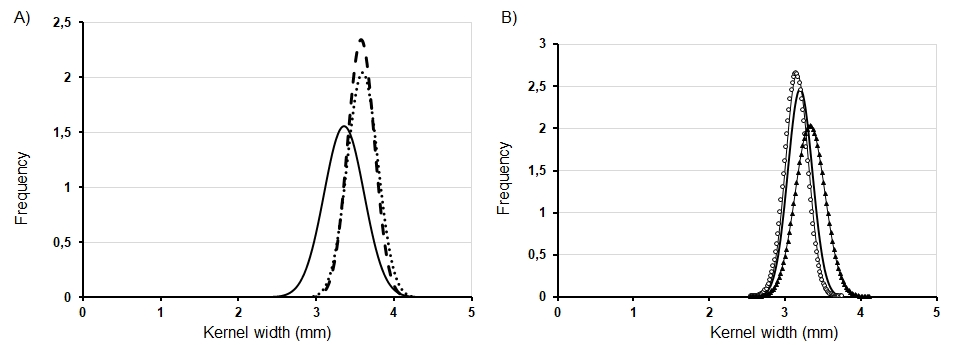
**

**Supplementary Figure S3.** Silencing barley *HvYrg* gene variants can result in wider kernels.

**A:** Distribution of kernel width parameters produced by plants from the *ASHvYrg1/1* genotype (**……**, x̄ = 3.59 mm), from the *ASHvYrg2/2* genotype (**- - -**, x̄ = 3.57mm) and from the GP plants (**___**, x̄ = 3.15 mm). **B.** Differences in width distribution of kernels produced by the PCR+ (N^o^ 7,
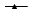
, x̄ = 3.33 mm), PCR- (N^o^ 8,
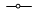
, x̄ = 3.15 mm) segregants from the *ASHvYrg1/1* line and GP plants (**___,** x̄ = 3.15 mm).
