# Supplementary material for: RING-Type E3 Ubiqitin Ligase Barley Genes (HvYrg1–2) Control Characteristics of Both Vegetative Organs and Seeds as Yield Components
Source: Plants (Basel). 2020 Dec 2;9(12):1693. doi: 10.3390/plants9121693 (PMC7761584; doi:10.3390/plants9121693)
Supplement: Supplementary file 1 [file plants-09-01693-s001.zip › Zip_supplementary/Supplementary Figure S1.rtf]

HvYrg1    1 MGNRIGGRR-KAGVEERYTRPQGLYEHRDIDQKKLRKLILETKLAPCYPGADDAAGADLE
HvYrg2    1 MGNRVGGRRRRPPVDERYTQPQGLYPHPDIDLKKLRRLILEAKLAPCHPGADDP-RPDLD


HvYrg1   60 ECPICFLYYPSLNRSKCCSKGICTECFLQMKPTHTARPTQCPFCKTPNYAVEYRGVKTKE
HvYrg2   60 ECPICFLFYPSLNRSKCCAKGICTECFLQMKSPTSCRPTQCPYCKMLNYAVEYRGVKTKE


HvYrg1  120 ERSIEQFEEQKVIEAQMRMRQQALQDEEDKMRRKQSRCSSSRTIAPTTEVEYRDICSTSY
HvYrg2  120 EKGVEQIEEQRVIEAQIRMRHQELQDDAERLKNKQVAASTDEVTTA--RV---EPCDTGG


HvYrg1  180 SAPPYRCTEQETECCSSEPSCSAQANM----RSFHSRHTRDGNIDMNIEDMMVMEAIWRS
HvYrg2  175 TSTPAASGAQ----GNDAPSCQVQHSELLLKNAERLRQLRDNNFDMDLEEVMLMEAIWLS


HvYrg1  236 IQEQGSIGNPACGSFMPFEQPTRERQAFVAASPLEIPHPGGFSCAVAAMTEHQPSSMDFS
HvYrg2  231 VQDQEALGNPGSIGAVPPTLPLRCYDAS-GATSAEAAPPGGFACAVAALAEQQHMLGDPS


HvYrg1  296 YMTGS--SAFPV----FDMFRRPCNIAGGSLRAVESSLDSWSGIAPSGTRREMVREEGEC
HvYrg2  290 SAATCQTSRHDILSRSQRSFTEDLSIAGSSSSAIRVEE------PPSNGRT--PQARDYS


HvYrg1  350 SIDHWSEGAEAGTSYAGSDIMADAGTMPPLP-FADNYSMAASHFRPESIEEQMMYSMAVS
HvYrg2  342 NNDGWSDVAEASTSCAGSDVTVEAGAASLAAAAASDVSSIGSGNVPDSFEEQMMLAMALS


HvYrg1  409 LAEAHGRTH-TQGLTWL
HvYrg2  402 LVDARGVGGSPPALAWR


Supplementary figure S1. The alignment of the amino acid sequences of two barley YRG proteins. The black frame shows the differences between their RING-domains.
