# Supplementary figures and images for: RING-Type E3 Ubiqitin Ligase Barley Genes (HvYrg1–2) Control Characteristics of Both Vegetative Organs and Seeds as Yield Components
Source: Plants (Basel). 2020 Dec 2;9(12):1693. doi: 10.3390/plants9121693 (PMC7761584; doi:10.3390/plants9121693)

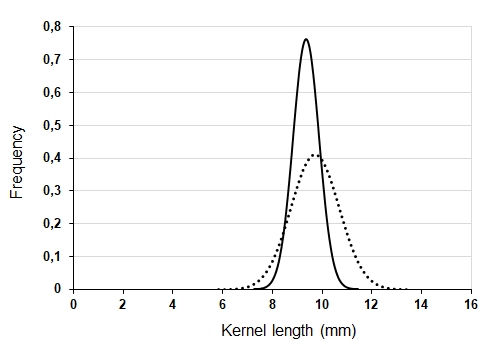

Supplement: Supplementary file 1 [file plants-09-01693-s001.zip › Zip_supplementary/Supplementary Figure S2.jpg]

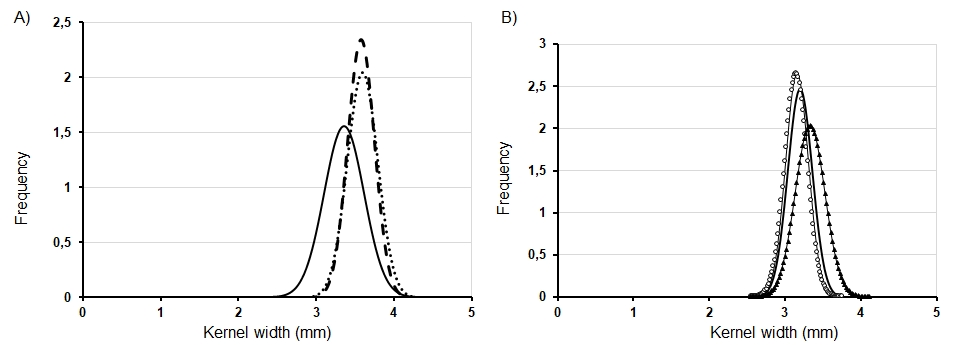

Supplement: Supplementary file 1 [file plants-09-01693-s001.zip › Zip_supplementary/Supplementary Figure S3.jpg]
